# An AI-powered data curation and publishing virtual assistant: usability and explainability/causability of, and patient interest in the first-generation prototype

**DOI:** 10.3389/fdgth.2025.1629413

**Published:** 2025-10-17

**Authors:** Rutger van Mierlo, Wenjie Liang, Kerli Norak, Michaela Kargl, Mall Maasik, Anne-Lore Bynens, Markus Plass, Markus Kreuzthaler, Martin Benedikt, Laura Hochstenbach, Arnoud van 't Hof, Remzi Celebi, Andre Dekker, Isabelle de Zegher, Petros Kalendralis, the AIDAVA consortium, Alexander Kreutz

**Affiliations:** ^1^ Department of Radiation Oncology (Maastro), GROW Research Institute for Oncology and Reproduction, Maastricht University Medical Centre+ (MUMC+), Maastricht, Netherlands; ^2^ Department of Cardiology, Cardiovascular Research Institute Maastricht (CARIM), Maastricht University, Maastricht, Netherlands; ^3^ Department of Cardiology, Maastricht University Medical Centre+ (MUMC+), Maastricht, Netherlands; ^4^ Department of Cardiology, Zuyderland Medical Centre, Heerlen, Netherlands; ^5^ Department of Health Technologies, Tallinn University of Technology, Tallinn, Estonia; ^6^ Diagnostic and Research Institute of Pathology, Medical University of Graz, Graz, Austria; ^7^ IT Department, North Estonia Medical Centre, Tallinn, Estonia; ^8^ Clinical Data Science, Maastricht University Medical Centre (MUMC+), Maastricht, Netherlands; ^9^ Institute for Medical Informatics, Statistics and Documentation, Medical University of Graz, Graz, Austria; ^10^ Department of Internal Medicine, Division of Cardiology, Medical University of Graz, Graz, Austria; ^11^ Department of Health Services Research, Care and Public Health Research Institute (CAPHRI), Faculty of Health Medicine and Life Sciences, Maastricht University, Maastricht, Netherlands; ^12^ Department of Advanced Computing Sciences, Maastricht University, Maastricht, Netherlands; ^13^ b!loba, Tervuren, Belgium

**Keywords:** data curation, interoperability, reusability, usability, explainability, causability, artificial intelligence, data publishing

## Abstract

**Introduction:**

Ensuring high quality and reusability of personal health data is costly and time-consuming. An AI-powered virtual assistant for health data curation and publishing could support patients to ensure harmonization and data quality enhancement, which improves interoperability and reusability. This formative evaluation study aimed to assess the usability of the first-generation (G1) prototype developed during the AI-powered data curation and publishing virtual assistant (AIDAVA) Horizon Europe project.

**Methods:**

In this formative evaluation study, we planned to recruit 45 patients with breast cancer and 45 patients with cardiovascular disease from three European countries. An intuitive front-end, supported by AI and non-AI data curation tools, is being developed across two generations. G1 was based on existing curation tools and early prototypes of tools being developed. Patients were tasked with ingesting and curating their personal health data, creating a personal health knowledge graph that represented their integrated, high-quality medical records. Usability of G1 was assessed using the system usability scale. The subjective importance of the explainability/causability of G1, the perceived fulfillment of these needs by G1, and interest in AIDAVA-like technology were explored using study-specific questionnaires.

**Results:**

A total of 83 patients were recruited; 70 patients completed the study, of whom 19 were unable to successfully curate their health data due to configuration issues when deploying the curation tools. Patients rated G1 as marginally acceptable on the system usability scale (59.1 ± 19.7/100) and moderately positive for explainability/causability (3.3–3.8/5), and were moderately positive to positive regarding their interest in AIDAVA-like technology (3.4–4.4/5).

**Discussion:**

Despite its marginal acceptability, G1 shows potential in automating data curation into a personal health knowledge graph, but it has not reached full maturity yet. G1 deployed very early prototypes of tools planned for the second-generation (G2) prototype, which may have contributed to the lower usability and explainability/causability scores. Conversely, patient interest in AIDAVA-like technology seems quite high at this stage of development, likely due to the promising potential of data curation and data publication technology. Improvements in the library of data curation and publishing tools are planned for G2 and are necessary to fully realize the value of the AIDAVA solution.

## 1 Introduction

Personal health data (PHD) consist of vast amounts of rich, structured and unstructured data in narrative forms, available in heterogeneous formats and scattered amongst healthcare systems. PHD is stored as hospital and non-hospital data in electronic health record (EHR) systems, which may or may not be interconnected (1–3). Curating and publishing PHD are costly and time-consuming, and consequently, PHD are difficult to reuse due to the large amounts of narrative text. For example, text-based content comprises 40%–80% of electronic health record information (3). These data could benefit healthcare and research if they are curated and published in an interoperable and reusable format for data users (i.e., patients or healthcare professionals).

In this article, “data curation and data publishing” denotes the integration, harmonization, and quality enhancement (data curation) of PHD, consisting of multimodal data, and its transformation into a target format (data publishing) to make these data more reusable for humans and machines. Today, expert data stewards can make sense of the unstructured data by using existing data curation tools, such as text mining and additional manual processing. However, due to the enormous amount of available PHD, parts of these data are undoubtedly not curated or unused, even though they represent a wealth of information for clinical care and clinical research (4). AI-based automated curation with an active human-in-the-loop (HITL) approach (5) could be a promising solution for data curation and data publishing.

Research has shown positive health-related outcomes, such as improved self-care or medication adherence, as a result of active patient engagement in managing their PHD (6). The problem, however, resides in enabling or motivating patients to actively engage in curating their PHD. Patients may prefer to take on a passive role, rather than an active role in entering or updating their PHD, especially with more complex medical information (6). Therefore, it seems unfeasible for some, if not most, patients to curate and enhance their PHD without adequate support from expert data stewards or AI and non-AI curation tools.

Currently, the approach to reuse PHD is population-centric curation (7), which relies on forms of mass curation by expert data stewards. PHD, anonymized or pseudonymized, cannot be linked across data sources. Moreover, further organizational (lack of skilled resources), cultural, ethical, and legal challenges remain prevalent (8). We believe a paradigm shift is required from population-centric and anonymized curation to individual-centric curation (7), supported by AI and non-AI curation tools and an HITL approach. Thus, multimodal patient data is transformed into a knowledge graph, which is defined as a semantic network that represents the relationships between entities or events in the real world (9). The sources of each patient’s data are curated into a source knowledge graph (SHKG); all SHKGs would then be integrated into a single personal health knowledge graph (PHKG) (9–11). PHKGs can be introduced to enhance the interoperability and reusability of PHD, provided they are supported by an ontology aligned with widely adopted standards and medical terminologies such as Systematized Nomenclature of Medicine Clinical Terms (SNOMED-CT) (12) and Logical Observation Identifiers Names and Codes (LOINC) (13). This would introduce the paradigm shift we believe necessary to provide a centralized, personal health dossier in an interoperable and reusable format (7).

The AI-powered data curation and publishing virtual assistant (AIDAVA) project (7, 14) that started in September 2022 aimed to support patients to quickly and automatically curate their PHD (15, 16) into a PHKG. In the first-generation (G1) prototype, we integrated existing and newly developed AI and non-AI data curation tools, with an intuitive front-end to support data curation and data publishing. We evaluated G1 in two separate, but relevant use cases. The first use case involved patients with cardiovascular disease (CVD), presenting a longitudinal health record. This includes hospital and non-hospital data collected across multiple organizations in heterogeneous formats, from which a Second Manifestations of Arterial Disease (SMART) risk score (17) could be calculated that primarily benefits clinical healthcare. The second use case involved patients with breast cancer (BC), addressing the issue of non-interoperable, cross-border patient registries and supporting international clinical research. The PHKGs from the patients in both use cases were extracted and visualized into a personal International Patient Summary (IPS), “an electronic health record extract containing essential healthcare information about a subject of care” following the emerging European Electronic Health Record Exchange Format (EEHRxF) standard identified in the European Health Data Space (EHDS) regulation (18). The aim of this formative evaluation study was to assess the usability and explainability/causability of (19) and patient interest in G1 in these use cases.

## 2 Methods

### 2.1 Research design

This formative evaluation study was conducted at four health institutions across three European countries. These were the North Estonia Medical Centre (NEMC) in Estonia, Maastro and the Maastricht University Medical Centre (MUMC+) in Netherlands, and the Medical University of Graz (MUG) in Austria. The patients tested G1 for at least 2–4 weeks, with support from the research team. The study flow for the patients is visualized in the Supplementary Materials. In addition, a list of the AI and non-AI tools deployed in G1 can be found in the Supplementary Materials.

### 2.2 Patient selection

The inclusion goal was set at 90 adult patients for adequate evaluation, equally dividing 30 patients between NEMC, Maastro/MUMC+, and MUG, each of which included 15 patients with BC and 15 patients with CVD, specifically with type 1 myocardial infarction. The recruitment period for G1 lasted from May 2024 to December 2024. The inclusion and exclusion criteria for G1 prototype testing are listed in Table 1. The development of G1 took place with the support of patient consultants selected by the European Patient Cancer Coalition (ECPC) (20) and European Heart Network (EHN) (21). They did not contribute their PHD but acted as patient representatives in the co-development of G1.

**Table 1:** Inclusion and exclusion criteria for G1 prototype testing.

Inclusion criteria	Exclusion criteria
Data available in the electronic health records within the related medical center	The patient was vulnerable, as judged by the physician
Owner and user of a smartphone	The patient was underage
Provide consent for the data curator, study nurse, and research associate to access and identify, and extract their PHD	
Sign a collaboration agreement with the relevant HDI, if applicable	
Agree to test both the G1 and G2 prototypes	
Understand and speak English or the local language (Dutch, Estonian, or German)	

### 2.3 Research setting

The start of the study was superseded by a “dry-run workshop”, conducted with the patient consultants in May 2024, as preparation for the evaluation of G1. A comprehensive training plan was developed, which included role specifications for study nurses, research associates, expert data curators, patients, and data users (clinicians) (Table 2). The patient consultants provided many valuable insights and feedback to implement before G1 testing that may have reduced the chance of errors during actual deployment.

**Table 2:** Task overview during G1 testing.

Role in G1	Main task(s)^a^
Study nurse and/or research associate	Extract PHD from EHR
	Fill in REDCap forms for the patients
	Contact for patients' concerns and questions
	Explain the ingestion, curation, publishing, and use steps to patients
	Administer questionnaires
Expert data curator	Supervise and support the ingestion, curation, publishing, and use steps for the patients
	Answer questions in AIDAVA if the patient has selected: “I don't know”
Patient	Work through the ingestion, curation, publishing, and use steps for G1 prototype testing
Data user/clinician	
BC specialist	Screening and recruitment
	Check the accuracy of BC registry inquiries
CVD specialist	Screening and recruitment
	Calculate the SMART risk score

^a^ The table provides a general overview of tasks within the research team. However, tasks could be interchangeable between each role if the appropriate skill and knowledge were present.

The dry run had three objectives, namely to (1) gather feedback on the AIDAVA prototype (pre-G1) at that stage and the health data intermediary (HDI) integration, (2) align the evaluation process across sites, and (3) consolidate feedback on the Research Electronic Data Capture (REDCap) tool. The patient consultants were asked to comment on the user journey, and the final draft is available in the Supplementary Materials. After the dry run, the formative evaluation started, and eligible patients were invited to an information session to explain the purpose of the study and go through the informed consent. Patients who signed informed consent forms were guided by the study nurse or research associate in a 1-h training session. In this session, the study nurse or research associate (1) reminded the patient of the objective of AIDAVA, (2) created an account with the patient for G1 and the HDI, and (3) explained the data flow and steps, as presented in Supplementary Figure S1.

**Figure 1:**
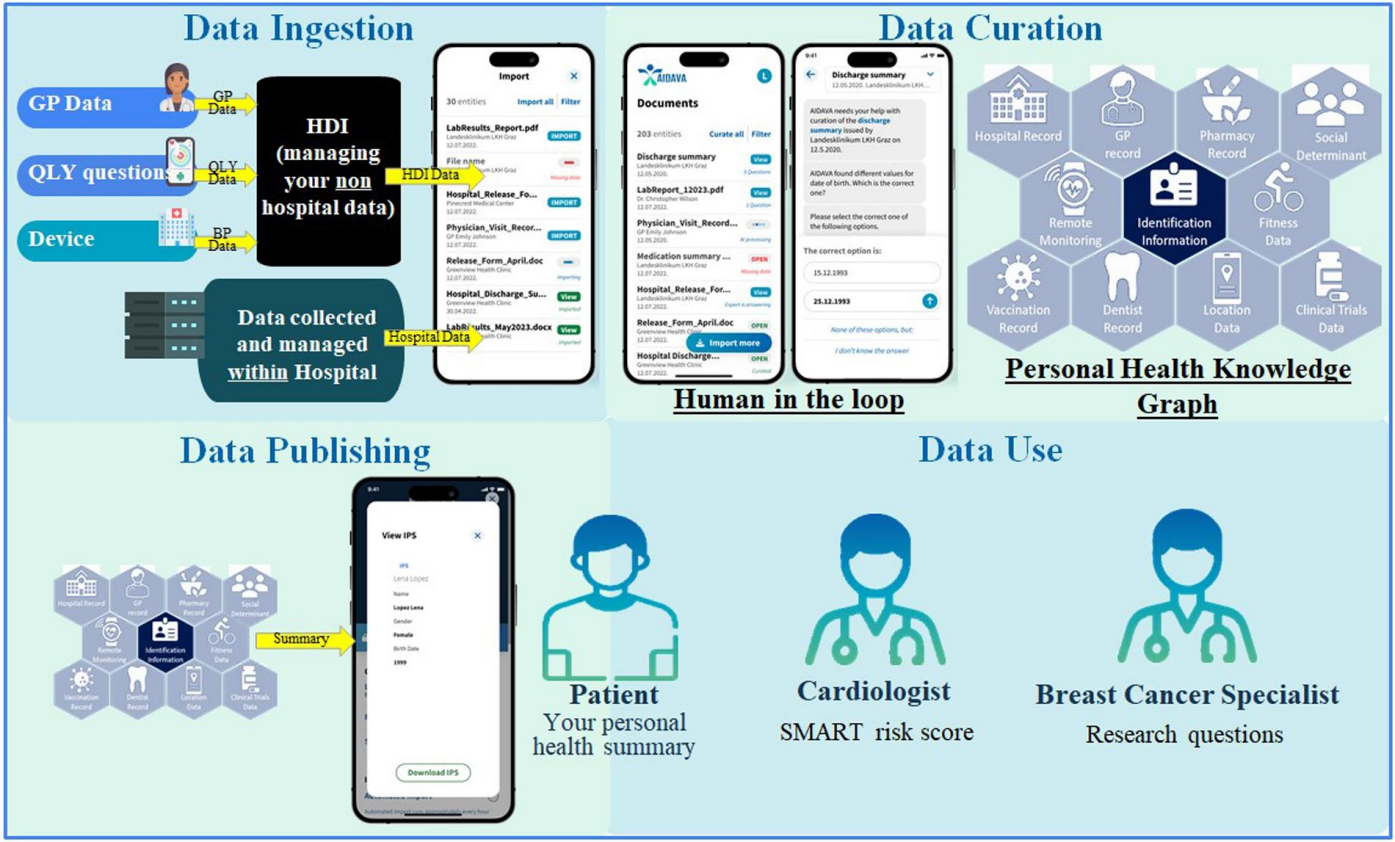
Overview of the data flow and steps. AI, artificial intelligence; BP, blood pressure; GP, general practitioner; HDI, health data intermediary; IPS, international patient summary; QLY, quality of life.

The patients worked through four steps (data ingestion, data curation, data publishing, and data use) after the deployment of G1 at the four health institutions acting as the test sites in this study (Supplementary Figure S1). The first three steps required active patient involvement, which include HITL mechanisms to improve the quality of the final IPS that the patient receives. In step 1, data ingestion, the patients consented to have their PHD identified and extracted from the hospital EHR, as well as from the HDI. In this context, HDI refers to an entity or platform that facilitates the collection, integration, and controlled sharing of PHD across different healthcare systems and data users. The patient actively connects their HDI account to the AIDAVA account. Then, these PHD were transferred to the AIDAVA data store, which was exclusively available within the hospital testing environment of the local site or a secure national cloud service. An overview of the complete data flow is illustrated in Figure 1.

In step 2, data curation, the patients activated their AIDAVA account to transform, integrate, and complete their ingested PHD into a standardized representation, the PHKG. As G1 is in the prototyping phase, the patients were warned that any output from the virtual assistant may be incorrect. G1 generated questions for the patients when complete automation of data curation failed, which was generally expected to occur due to missing data. This was the main role and implementation of the HITL mechanism, i.e., to catch any errors prior to data publication. If the patients were unsure of the answer or the question generated by G1 did not make sense to them, they were able to forward the question to the expert data curator appointed by the local health institution.

In step 3, data publishing, the data to be extracted from the PHKG were specified. For this formative evaluation study, three data publishing outputs were defined: (1) extraction and visualization of the patient's IPS, one of the six critical data categories proposed in the EHDS and compliant with the international Health Level Seven Fast Healthcare Interoperability Resources (HL7 FHIR) guideline (18); (2) extraction of data elements to create local BC registries at each site (BC use case); and (3) extraction of key variables supporting the automatic calculation of the patient's SMART risk score (17) (CVD use case). These data elements and key variables can be found in the Supplementary Materials. The curated PHD, which are the source of these three different outputs, only need to be curated once as the respective data extraction processes are executed multiple times in the PHKG.

In step 4, data use, these three outputs were exploited by different data users (Figure 1). The patients were able to visualize their IPS through a specific visualization tool (MIDATA IPS Viewer). The BC specialist could access the metrics calculated from their local BC registry and from interoperable cross-border registries. The prototype executes federated queries for the virtual cross-border BC registry to avoid any data transfer across hospitals. The CVD specialist could visualize the automatically calculated SMART risk score and the details of its calculation for each patient. Due to the possibility of inaccurate data curation at this prototype stage, the SMART risk scores were solely used to test the prototype's accuracy, not for communicating actual risk to the patients.

The patients began testing G1 under the guidance of their local study nurse and/or research associate. Each patient tested and evaluated G1 for a duration of 2–4 weeks, starting with an on-site visit in which the study nurse supported the patient with the activation of their AIDAVA account. In addition, the patients created a personal HDI account, filled out the Medical and Digital Literacy questionnaires, and created an account for their blood pressure device (only for patients with CVD).

As shown in Figure 1, there were two paths for uploading data into the patient's AIDAVA account. First, after the patient had signed the informed consent form, the study nurse/research associate extracted the PHD of the patient from the hospital EHR and imported these data into the patient's personal AIDAVA account. Second, the patients uploaded PHD to their HDI account (including data from their blood pressure device), which were also sent to the patient's personal account within the hospital’s secure environment setup for AIDAVA, respecting the security requirements of each institution. In the remaining weeks, the patients tested the system with general support from the study nurse/research associate, and technical support from the expert data curator, who used a shared ticketing system to quickly solve any issues.

Once the data from different sources, typically in a heterogeneous format, were ingested, the patient could then request the AIDAVA system to curate them into a PHKG. This structured representation of PHD is compliant with the AIDAVA ontology based on the Swiss Personal Health Network (SPHN), including the SNOMED, LOINC, and FHIR profiles and is easily mappable to multiple standards, supporting semantic interoperability. AIDAVA uses an AI-powered semantic transformation infrastructure to orchestrate curation workflows; it leverages predefined data source descriptions (i.e., dictionaries) to process and transform data based on its meaning, rather than just its format. Each attribute/column in (semi-)structured data sources is first semi-automatically mapped either to the AIDAVA ontology with classical extract, transform, and load (ETL) transformation tools, or to dedicated curation tools such as entity linking (mapping to clinical terminologies) and entity alignment (linking terms from two different terminologies), supporting transformation of the data source into an SHKG compliant with the AIDAVA ontology. Unstructured data were directly processed by the natural language processing (NLP) tools and transformed into another SHKG. When data from each SHKG are integrated into a single patient's PHKG, the entity deduplication tool removes duplicate records referring to the same real-world entity and validation is performed using individual data quality checks. Quality scores are computed across multiple dimensions (e.g., completeness and consistency) and categories (e.g., valid code and temporal order consistency) and each detected quality issue is formulated as a question to the user to support data improvement. The entire curation and quality enhancement process is recorded within an audit trail to meet regulatory compliance.

The main task for the patients was to test data ingestion and data curation of their PHD, while checking the accuracy of the published PHD in their own IPS (Figure 1). More precisely, this meant that patients ingested files and documents that were uploaded to their personal AIDAVA account, so that these files and documents would then be available to them for automatic curation. To preserve data ownership, the patients could always choose whether to start automatic data curation for all or only some of the ingested files. G1 addressed questions to the patient whenever issues during the automatic curation arose (i.e., missing data, incompatible formats, unrecognizable documents, etc.). The patients answered these questions in a format related to the question (i.e., when asked for a date, they were presented with a “mm/dd/yyyy” format input field). The patients always had the possibility to either answer the question if they knew the answer or skip the question by clicking the “I don't know the answer” option. When patients selected the “I don't know the answer” option, the question was automatically forwarded to the expert data curator. To finalize the question, the patients either provided feedback on the question or pushed the “skip feedback” button. Furthermore, the patients were always able to check their IPS. At the closing evaluation session, the patients provided feedback on each step in the evaluation study, and the patients’ comments were documented in REDCap forms (22, 23) by the study nurse and/or research associate.

### 2.4 Data collection

Evaluation data were collected via REDCap forms (questionnaires and narrative feedback) by the study nurse and/or research associate. The patients' answers to the Medical and Digital Literacy questionnaire were collected during the first on-site visit. The patients' answers to the G1 evaluation questionnaires were collected after testing the prototype for 2–4 weeks after the first on-site visit. These questionnaires included the system usability scale (SUS) (24) and study-specific questionnaires on the explainability/causability of G1 and the patient's interest in AIDAVA-like technology.

The patients were asked to comment on the questionnaires, the HDI, the blood pressure device, and each of the four deployment steps of G1 (data ingestion, data curation, data publishing, and data use). These comments were collected in the REDCap forms. The patients were given a time sheet to track how much time they spent on testing G1, and how much time they spent on study-related activities other than G1 testing (which include blood pressure measurements and other study-related activities).

### 2.5 Data analysis

All the quantitative data from the questionnaires are presented as mean ± standard deviation, median, and range. The study-specific Medical and Digital Literacy questionnaires contained six questions in each domain. The answers ranked from 0 (i.e., no knowledge) to 5 (i.e., expert knowledge). The purpose of the Medical and Digital Literacy questionnaires was user profiling, so that in an ideal situation, the virtual assistant could adjust communication to the patient accordingly. The evaluation questionnaires comprised the SUS questionnaire, which contains 10 questions, six study-specific questions on explainability/causability, and six questions related to the patient's interest in AIDAVA-like technology. The answers to these questions ranged from 1 (strong disagreement) to 5 (strong agreement) on a 5-point Likert scale. The item responses for each of the three sets of SUS questionnaires were analyzed using cumulative link mixed models, including a random intercept per patient. The Cronbach's alpha values were used to assess the reliability of the SUS questionnaires. The original 10 SUS questions were used to calculate the SUS score (0–100), which was then correlated to a level of acceptability and net promoter score (25). Data on time spent on data ingestion and data curation are presented as mean ± standard deviation, median, and range. Comparisons between the two use cases (BC vs. CVD) and questionnaire items were analyzed using independent-samples tests (*t*-test or Mann–Whitney *U* test, as appropriate). A *P*-value of 0.05 or less was considered statistically significant in all the analyses.

### 2.6 Ethics statement

The study was approved by each local ethics committee at the participating test sites. An ethical advisory board, which includes external advisors, oversaw the G1 development process and the dry run workshop to ensure the study met all ethical and procedural standards.

## 3 Results

### 3.1 Participants

A total of 423 patients were screened for inclusion in the study, with 182 for the CVD use case and 246 for the BC use case (Table 3). A total of 83 patients signed informed consent forms for G1 testing. However, 13 patients withdrew before or during the testing of G1. The reasons for withdrawals were lack of motivation, difficulty using digital devices, personal reasons, the perceived effort required for testing G1, or a combination of these. Ultimately, 70 patients successfully tested G1.

**Table 3:** Overview of the patients screened and recruited, the withdrawals, and the number finalized per site.

Study stage	CVD use case				BC use case			
	NEMC	MUG	MUMC	Total	NEMC	MUG	Maastro	Total
Screened	32	110	40	182	35	142	246	423
Recruited—signed informed consent	13	10	15	38	15	15	15	45
Withdrawals^a^	4	3	1	8	1	4	0	5
Finalized	9	7	14	30	14	11	15	40

^a^ Withdrawals after signing informed consent.

### 3.2 Medical and digital literacy

The patients reported significantly higher mean digital literacy scores (18.2 ± 6.7) than medical literacy scores (15.6 ± 5.9) (*P* = 0.01) (Table 4). Moreover, the patient-reported medical literacy scores were significantly higher for the patients in the BC use case (17.3 ± 5.9) compared to those in the CVD use case (13.3 ± 5.0) (*P* < 0.01). There was no significant difference in the patient-reported digital literacy scores between the BC use case (18.1 ± 6.3) and the CVD use case (18.3 ± 7.1) (*P* = 0.92).

**Table 4:** The patient-reported medical and digital literacy and system usability scores for G1.

Questionnaire	Mean ± SD	Median	Range
Medical literacy (score 0–30) (*n* = 70)	15.6 ± 5.9	15.0	2.0–30.0
BC (*n* = 40)	17.3 ± 5.9	17.0	5.0–30.0
CVD (*n* = 30)	13.3 ± 5.0	14.0	2.0–22.0
Digital literacy (score 0–30) (*n* = 70)	18.2 ± 6.7	19.0	2.0–30.0
BC (*n* = 40)	18.1 ± 6.3	18.5	7.0–27.0
CVD (*n* = 30)	18.3 ± 7.1	20.0	2.0–30.0
System usability scale (score 0–100) (*n* = 62)	59.1 ± 19.7	57.5	15.0–97.5
BC (*n* = 34)	61.5 ± 18.0	61.3	15.0–95.0
CVD (*n* = 28)	55.9 ± 20.9	55.0	22.5–97.5

### 3.3 System usability

**Figure 2:**
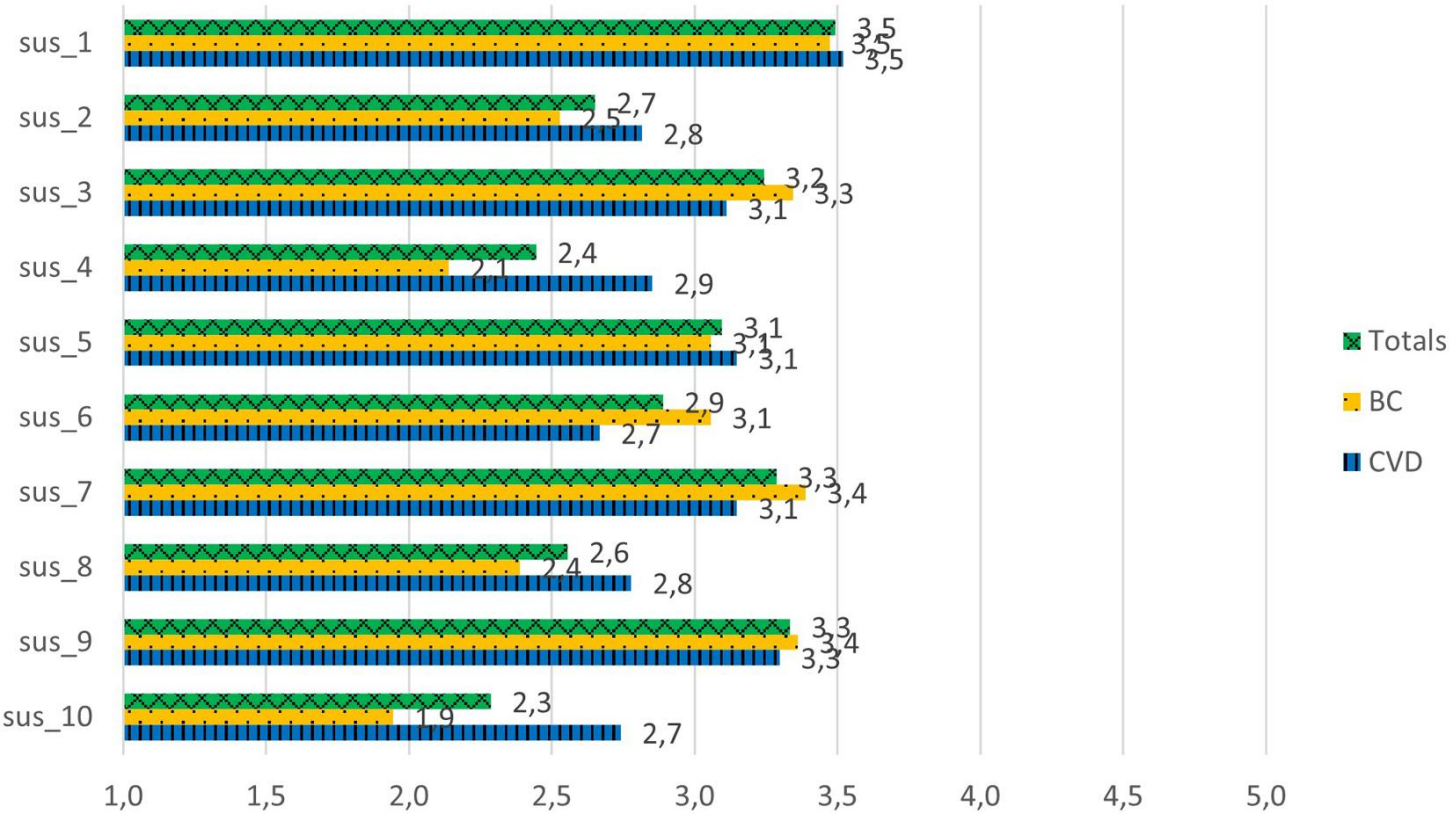
System usability scale scores for the G1 prototype*.* sus_1: I am ready to use this system frequently. sus_2: I found the system unnecessarily complex. sus_3: I thought the system was easy to use. sus_4: I would need the support of a technical person to be able to use this system. sus_5: I found the various functions in this system were well integrated. sus_6: There was too much inconsistency in this system. sus_7: Most people would learn to use this system very quickly. sus_8: I found the system very awkward to use. sus_9: I felt very confident using the system. sus_10: I needed to learn a lot of things before I could get going with the system.

The results suggest that the usability of G1 was marginally acceptable to the patients, as the mean score of the total was 59.1 ± 19.7 (Table 4) (Cronbach's *α* = 0.85). In total, 18 patients scored G1 higher than 68, which was considered the threshold of complete acceptability. In contrast, 18 patients scored G1 lower than 52, which means one should consider them detractors. The statement that scored the highest was “I am ready to use this system frequently”, with an average score between neutral and agreement (3.5 out of 5) (Figure 2). The lowest scores were for the statements “I would need the support of a technical person to be able to use this system”, “I found the system very awkward to use”, and “I needed to learn a lot of things before I could get going with the system”, with average scores between disagreement and neutral (2.3–2.6 out of 5). The comments by patients ranged from acceptance to rejection, as evidenced by the following two quotes:

“AIDAVA is a system you can get used to, but it needs time and training. As soon as you understand it, there is nothing complicated.”

“In general, AIDAVA G1 is not reasonably usable for the average user, cannot be recommended. The main basis for this conclusion is the inability to obtain or analyze sufficiently high-quality data. If one of the doctors starts making decisions based on a health report synthesized from low-quality data, it can be a threat to human life.”

### 3.4 Explainability/causability

**Figure 3:**
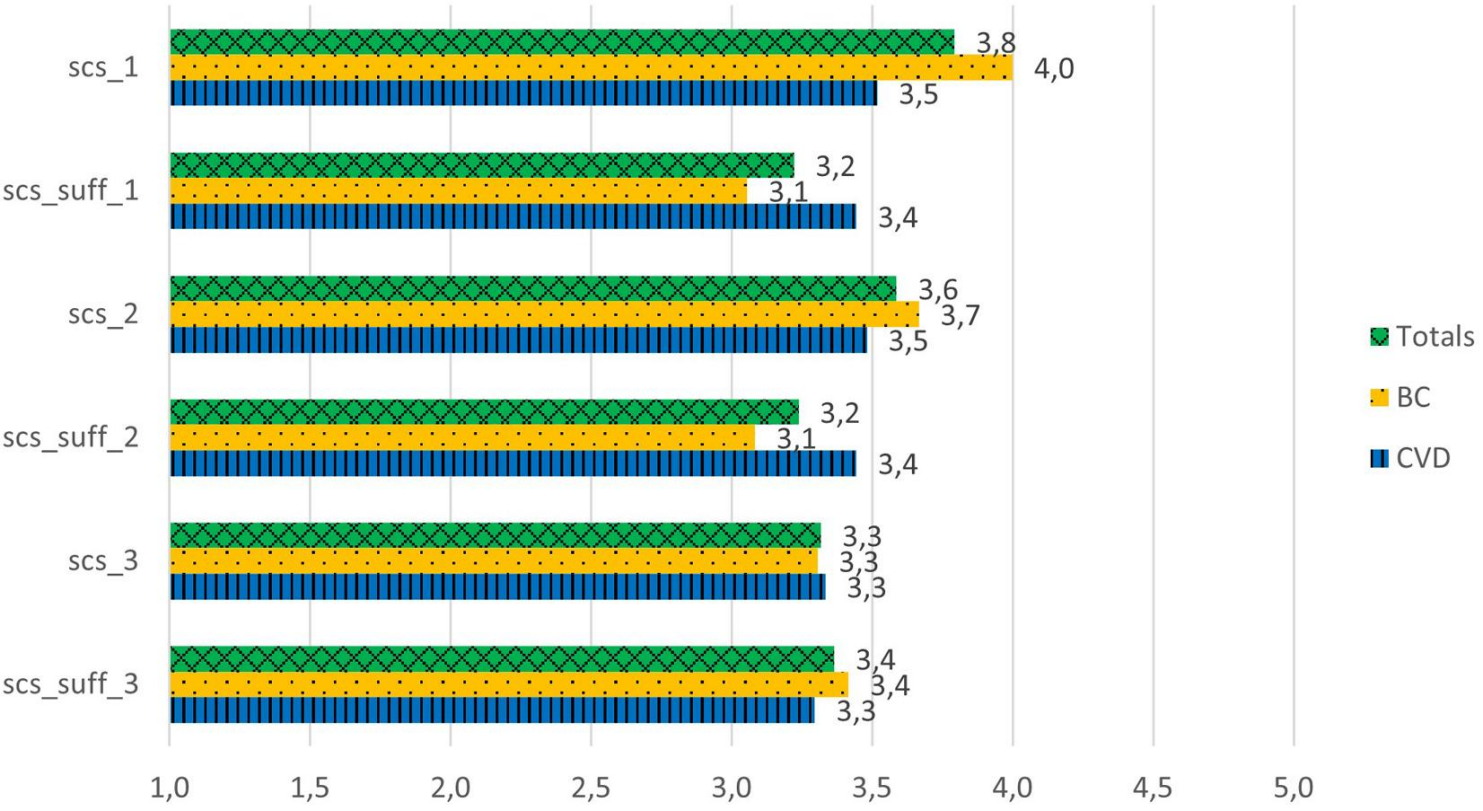
Explainability/causability scores for the G1 prototype*.* scs_1: For me, it is important to know where the different curated health data are coming from. scs_suff_1: in my opinion, information regarding this aspect is sufficient in AIDAVA. scs_2: For me, it is important to know who has curated my health data and which tools have been used. scs_suff_2: In my opinion, information regarding this aspect is sufficient in AIDAVA. scs_3: For me, it is important to know whether a health data curation method has been applied by a human or an algorithm. scs_suff_3: In my opinion, information regarding this aspect is sufficient in AIDAVA.

The patients had moderately positive scores for the explainability/causability questions, scoring between 3.3 and 3.8 out of 5 (Figure 3) (Cronbach's *α* = 0.69). The results indicate a significant difference between the scoring for the question “For me it is important to know where the different curated health data are coming from” and the scoring of the question related to its explainability in AIDAVA, “In my opinion, information regarding this aspect is sufficient in AIDAVA” (3.8 vs. 3.2; *P* = 0.01). The patients in both the BC and CVD use cases agreed in their scoring of explainability/causability. A specific comment by a patient in the CVD use case emphasized the lack of explainability as to where the PHD came from:

“What does ‘medical partner’ mean under file names? This should mention WHERE the document came from. That is unclear with this term.”

### 3.5 Patient interest

**Figure 4:**
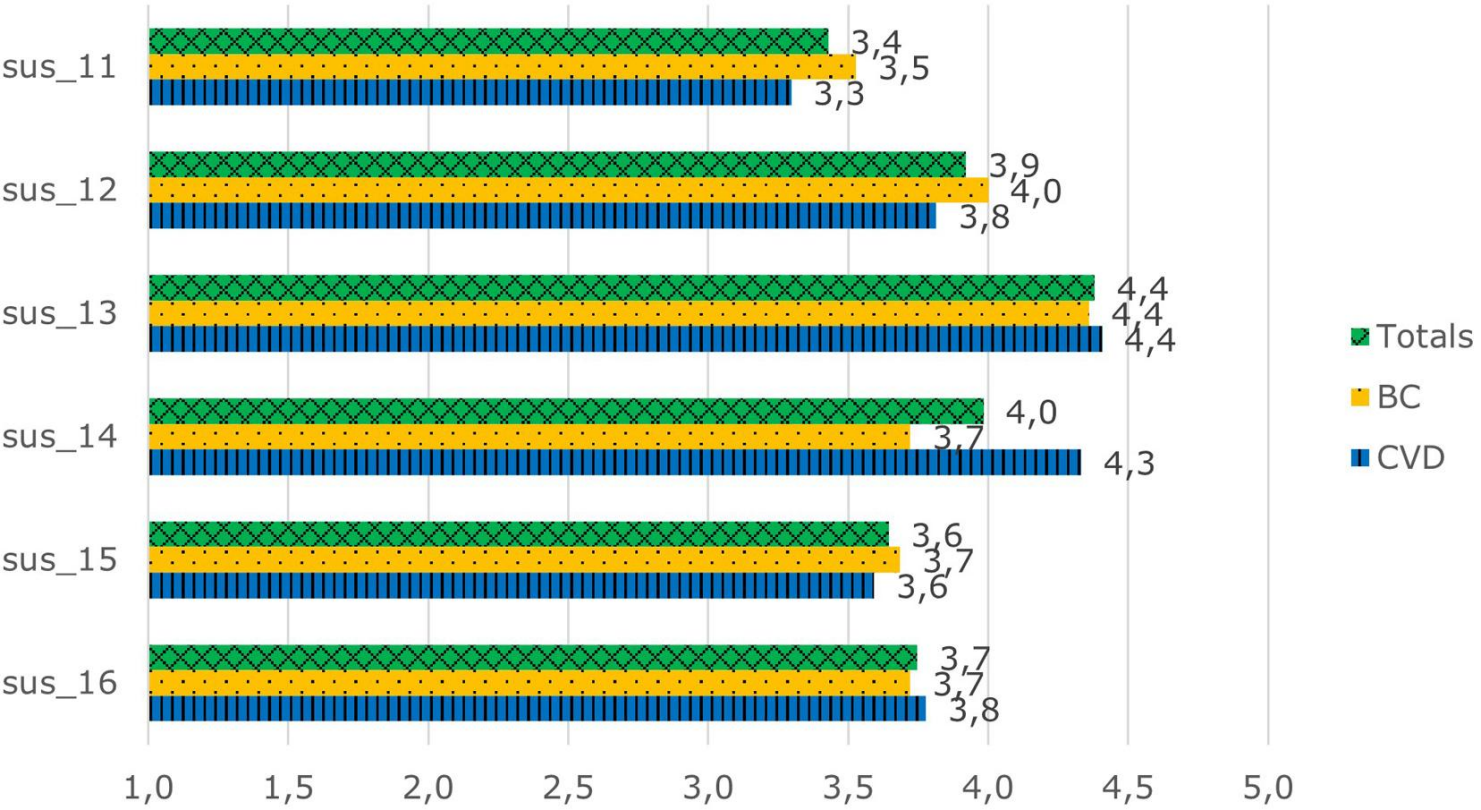
Overall interest scores in AIDAVA-like technology*.* sus_11: I would recommend AIDAVA to my friend, colleague, or family member. sus_12: I am ready to work with AIDAVA when it is available on the market. sus_13: I understand the purpose of data curation. sus_14: I am ready to spend the needed time to ensure proper data curation of my data. sus_15: This system is unique and different from anything else available. sus_16: This system will allow me to manage my health records better.

The patients were moderately positive to positive in their interest in AIDAVA-like technology, scoring between 3.4 and 4.4 out of 5 (Figure 4) (Cronbach's *α* = 0.78). The patients in both the BC and CVD use cases were in agreement in their scoring, except for the question “I am ready to spend the needed time to ensure proper data curation of my data”, as the patients in the CVD use case were more willing to spend time to complete data curation than the those in the BC use case (4.3 vs. 3.7; *P* = 0.012).

### 3.6 Prototype testing

The time spent on G1 consisted of “data ingestion and data curation” and “other activities,” such as the training, visit(s), or visualization of PHD. On average, the patients spent 124 ± 132 min on data ingestion and data curation, with a median of 75 min and a range of 0–635 min (Table 5). The patients in the BC use case spent significantly less time on average on data ingestion and data curation than those in the CVD use case (78 vs. 176 min, *P* < 0.01). The patients spent 78 ± 37 min on average on other activities related to G1 testing, with no significant differences between the BC and CVD use cases (78 ± 35 vs. 78 ± 38, *P* = 0.99).

**Table 5:** Patient-reported time (min) spent on G1 testing.

Activity	Mean ± SD	Median	Range
Data ingestion and data curation (*n* = 60)	124 ± 132	75	0–635
BC (*n* = 32)	78 ± 62	60	5–290
CVD (*n* = 28)	176 ± 166	130	0–635
Other activities	78 ± 37	80	0–175
Training, visit(s), and visualization (*n* = 55)			
BC (*n* = 32)	78 ± 35	80	19–175
CVD (*n* = 23)	78 ± 38	75	0–150

## 4 Discussion

The aim of this formative study was to evaluate G1’s usability and explainability/causability and patient interest in AIDAVA-like technology. It allowed us to assess the AI-powered automatic health data curation and data publishing workflow, gather feedback regarding its strengths and weaknesses, and identify areas for improvement and necessary changes that need to be considered for the development of second-generation (G2) prototype. Before testing using real patient data, the system was built and tested using synthetic test data, which, although it performed sub-optimally, was considered adequate by the evaluation site to proceed with this formative evaluation. Despite its perceived marginal acceptability among the patients who tested it using real-world data, G1 shows potential in automating data curation into a PHKG.

Apart from well-established tools, such as the optical character recognition (OCR) tool TESSERACT (26) and the German NLP tool AVERBIS Health Discovery (27), the majority of the curation tools integrated into G1 were still in the early stages of development and had not yet been fully tested and refined. However, they offered a promising solution and were seen as a better alternative to not having these tools in G1. For example, to extract structured data from Estonian and Dutch text, the AIDAVA consortium is developing a multi-lingual model capable of extracting concepts directly from these languages; this tool will be available in G2. In G1, we had to use a translation tool to translate these languages into German and then use the AVERBIS tool. This could have resulted in suboptimal translation and extraction. Unfortunately, this has likely contributed to the marginal acceptability of G1 but leaves an opportunity for improvement after further development.

Regarding the perceived usability of G1 by the patients, there was an equal mix of complete acceptability and detraction (approximately 25% each), according to Brooke’s scoring system (28). The fact that this was a formative evaluation study likely explains some of the detraction at this stage of development. The unfinished integration of all the curation and publishing tools could give patients a sense of it being an early prototype (which G1 is). Conversely, acceptability at this stage may be explained by the perceived future value of AIDAVA-like technology. Our data on the overall interest in AIDAVA-like technology suggests as much, as the patients’ scores for these questions were moderately positive to positive. As for the explainability/causability of data curation in G1, there were specific aspects that required improvement. For example, there was a discrepancy in scores between the questions “For me it is important to know where the different curated health data are coming from” and its follow-up “In my opinion, information regarding this aspect is sufficient in AIDAVA”. This illustrates a clear need for explainability from the patients' point of view. An explainability module incorporated in G2 may address this issue.

Overall, the user interface of G1 was considered quite straightforward and easy to use. Some patients suggested implementing a push notification when documents were ready to be ingested, as patients would have to manually check if documents were ready or they would have someone from the research team notify them, which they considered to not be user-friendly. Patients who curated documents found the procedure easy to follow and the patients who uploaded documents via their HDI found this to be very convenient and straightforward. Overall, G1 was, as expected, no more than marginally acceptable to the patients. The patients scored the lowest for the question, “Would you recommend AIDAVA to a friend, colleague, or family member?” at this stage of the development. Conversely, the patients scored highly in “understanding the purpose of data curation.” This suggests that the patients saw the potential for a well-developed, AI-supported virtual assistant that ingests and curates their PHD, which G1 has not achieved thus far due to its early development stage.

In addition, comments were made about the HITL dialogue, which will be improved in G2. Full automation without errors or missing data did not occur in any of the patients' PHD. The goal of the HITL dialog was to provide the patients with the correct context when asking for the missing data. However, the communication between the intelligent virtual assistant and the patient was not understandable in many cases and lacked the very context that would have been necessary for understandability. Comments such as “[t]he questions were not asked in a simple way/in simple language” were reiterated by patients across sites in both use cases. An example of the language used is as follows: “AIDAVA needs your help with patient identification (admission, discharge, transfer information). Information about hat Geburtsdatum is missing” [original question for the Dutch patient: “AIDAVA heeft uw hulp nodig bij patient identification (admission, discharge, transfer information). Informatie over hat Geburtsdatum mist”]. Even though patients could understand that a date of birth is being required from the context, the terms “admission, discharge, transfer information,” referring to the source documents, were not understandable for the average patient. In addition, the German words scattered through the question for non-German patients gave a strong sense of G1 being an early prototype, which may also have negatively impacted usability scores. Therefore, implementing the complete set of curation tools for G2 will likely benefit the HITL approach and will likely result in more favorable usability and patient interest outcomes.

In the development of G2, the AIDAVA consortium will focus on improving and integrating the curation tools according to the findings from the G1 evaluation. A critical component of the configuration and deployment of G1 was the technical specification of the data to be exchanged between the hospital system and G1. The data transfer specification (DTS) was designed to ensure the consistency and accuracy of the data transfer. Unfortunately, during the deployment of G1 in the Netherlands, the DTS was not properly configured, which led all the patients with BC and a few patients with CVD to have issues with data ingestion and curation. Even though the issue of data ingestion was solved in time, curation was solved too late for these patients, likely impacting the acceptability of G1. Thus, the AIDAVA technical team will explore and develop solutions to facilitate necessary configurations for this approach and ensure that the automated data curation and data ingestion workflow will be streamlined and effective in G2.

In addition, further development of the publishing tools could provide patients with the most tangible use for their PHD (and PHKG), in the form of their IPS. Unfortunately, due to the incomplete integration of the curation tools as described before and the resulting incomplete quality of the PHKG, data publishing was not well covered by G1. Thus, the evaluation of the publishing step will be prioritized in the G2 assessment. Moreover, we aim to upgrade the entire automated workflow for effective data curation and data publishing by smoothing the integration of both non-AI and AI-based tools. We will improve the HITL process based on large language models and optimize the human–machine interaction according to the users' medical and digital literacy.

## 5 Conclusion

Despite its marginal acceptability, G1 shows potential in automating data curation into a personal health knowledge graph, but it has not reached full maturity yet. G1 was intended to reuse existing curation tools. However, apart from a few off-the-shelf software solutions, there were no suitable tools available for reuse. As a result, the team had to rely on very early prototypes of tools that were originally planned for use in G2. This may have contributed to lower usability and explainability/causability. Conversely, patient interest in AIDAVA-like technology seems quite high at this stage of development, likely due to the promising potential of curation and publication technology. Improvements in the library of curation and publication tools are planned for G2 and are necessary to fully realize the value of the AIDAVA solution.

## Data Availability

The datasets presented in this article are not readily available because the consortium project is not finished at the time of publication. Requests to access the datasets should be directed to rutger.vanmierlo@maastrichtuniversity.nl.

## Group members of the AIDAVA consortium

Alexander Kreutz, Bern University of Applied Sciences, Biel, Switzerland; Alpár Tana, Egnosis, Sfantu Gheorghe, Romania; Aranka Ravai-Nagy, Egnosis, Sfantu Gheorghe, Romania; Bart Scheenstra, Department of Cardiothoracic Surgery, Heart and Vascular Centre, Maastricht University, Maastricht, the Netherlands; Béla Bihari, Egnosis, Sfantu Gheorghe, Romania; Botond Kiss, Egnosis, Sfantu Gheorghe, Romania; Dominik Steiger, MIDATA Cooperative, Zurich, Switzerland; Elion Sõber, North Estonia Medical Centre, Tallinn, Estonia; Emmanuel Benoist, Bern University of Applied Sciences, Biel, Switzerland; Eno-Martin Lotman, North Estonia Medical Centre, Tallinn, Estonia; Erol Ensar, Maastricht University Department of Advanced Computing Sciences, Maastricht, Netherlands; Gleb Klimenkov, North Estonia Medical Centre, Tallinn, Estonia; Gregorio Sambataro, European Heart Network, Brussels, Belgium; Heimo Müller, Medical University of Graz, Graz, Austria; István Horváth, Egnosis, Sfantu Gheorghe, Romania; János Dániel Dallos, Egnosis, Sfantu Gheorghe, Romania; Katerina Zdravkova Serafimova, ONTOTEXT AD, Sofia, Bulgaria; Katrin Lepik, North Estonia Medical Centre, Tallinn, Estonia; Kertu Mõistlik, North Estonia Medical Centre, Tallinn, Estonia; Kristian Kankainen, North Estonia Medical Centre, Tallinn, Estonia; Liesbeth Boersma, Maastricht University Medical Centre+, Maastricht, Netherlands; Lóránt Ferencz, Egnosis, Sfantu Gheorghe, Romania; Michel Dumontier, Department of Advanced Computing Sciences, Maastricht University, Maastricht, Netherlands; Monika Moga, Egnosis, Sfantu Gheorghe; Natalja Šermolajeva, North Estonia Medical Centre, Tallinn, Estonia; Sina Amirrajab, The D-Lab, Department of Precision Medicine, GROW—Research Institute for Oncology and Reproduction, Maastricht University, Maastricht, Netherlands; Svetla Boytcheva, Ontotext, Sofia, Bulgaria; Todor Primov, Ontotext, Sofia, Bulgaria; Zoltán Lázár, Egnosis, Sfantu Gheorghe, Romania; Zsolt Máté, Egnosis, Sfantu Gheorghe, Romania.
