# Supplementary material for: An AI-powered data curation and publishing virtual assistant: usability and explainability/causability of, and patient interest in the first-generation prototype
Source: Front Digit Health. 2025 Oct 17;7:1629413. doi: 10.3389/fdgth.2025.1629413 (PMC12576891; doi:10.3389/fdgth.2025.1629413)
Supplement: Supplementary file 1 [file Datasheet1.pdf]

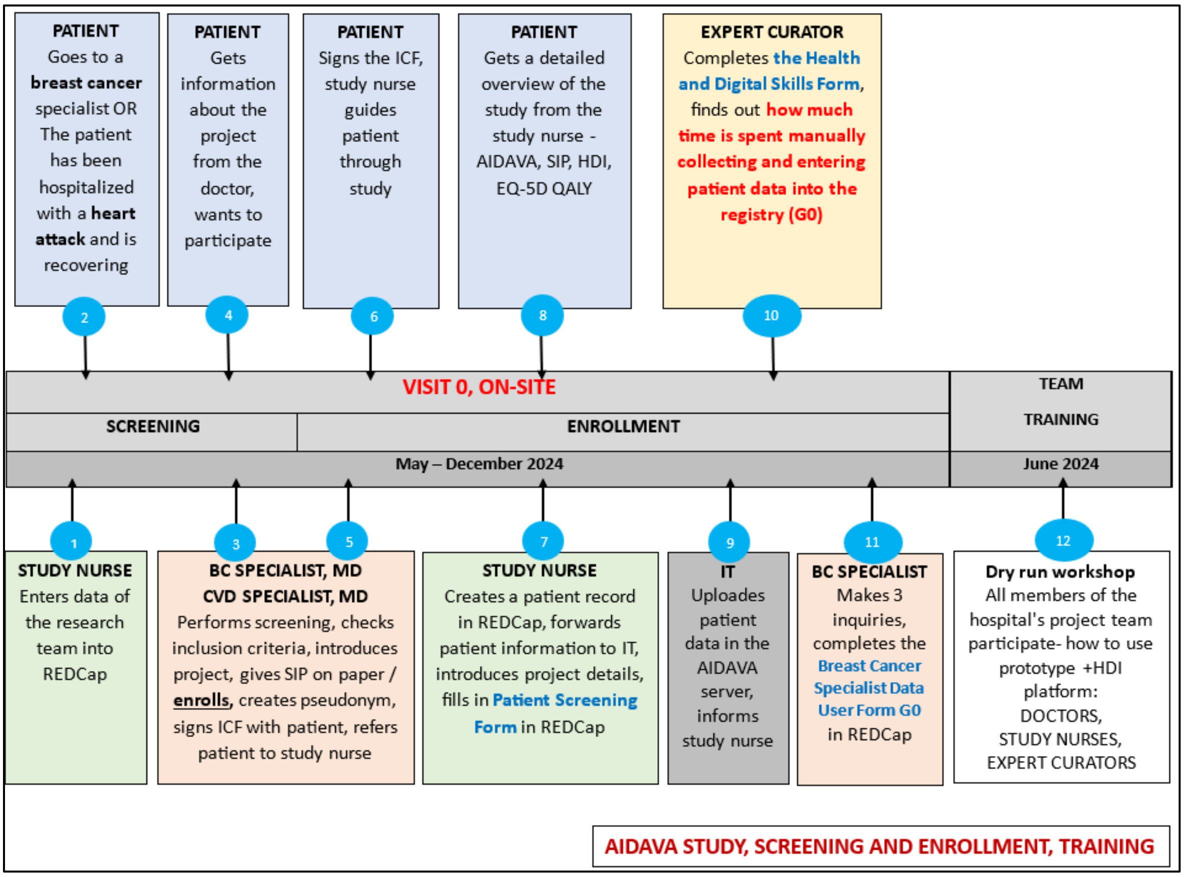

Supplementary Figure 1. *Description of the different steps to test the system with patients (Screening, enrolment and team training)*

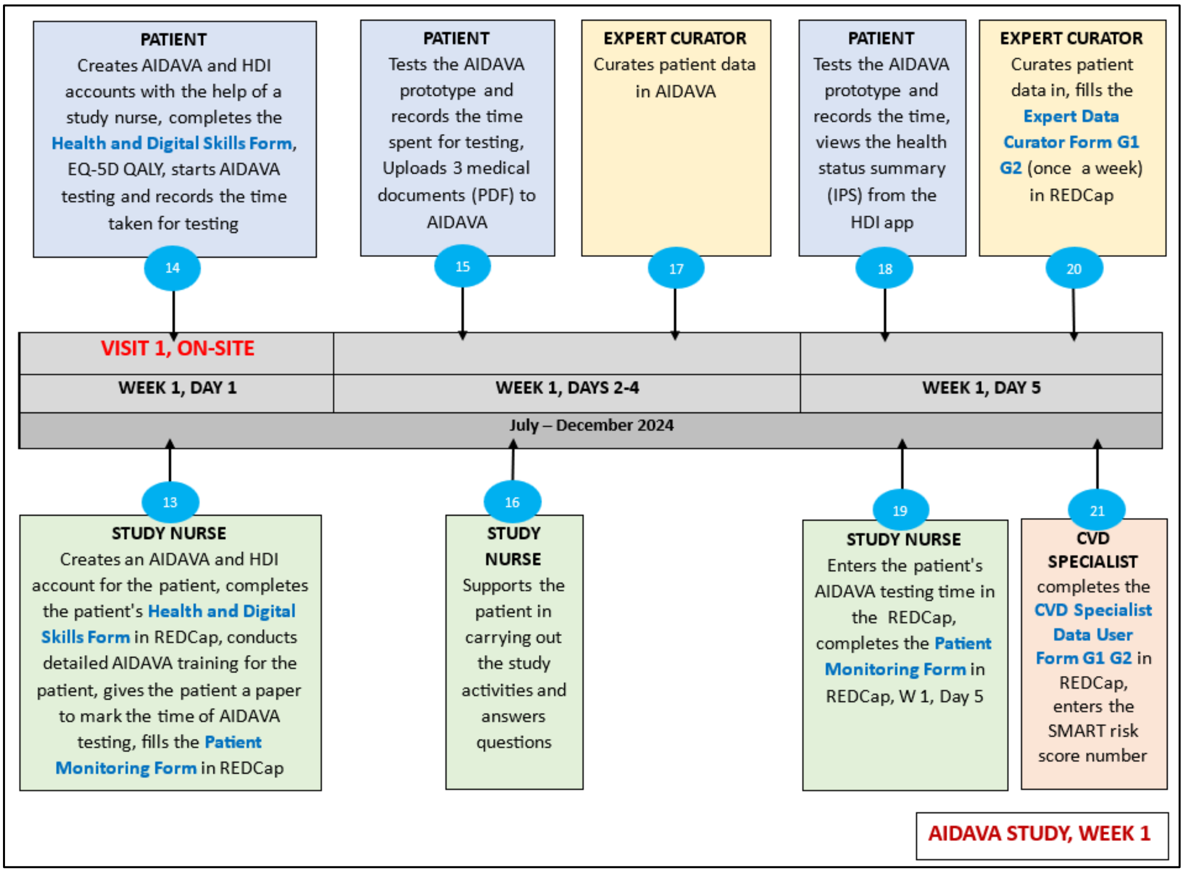

Supplementary Figure 2. *Description of the different steps to test the AIDAVA system with patients (Weeks 1)*

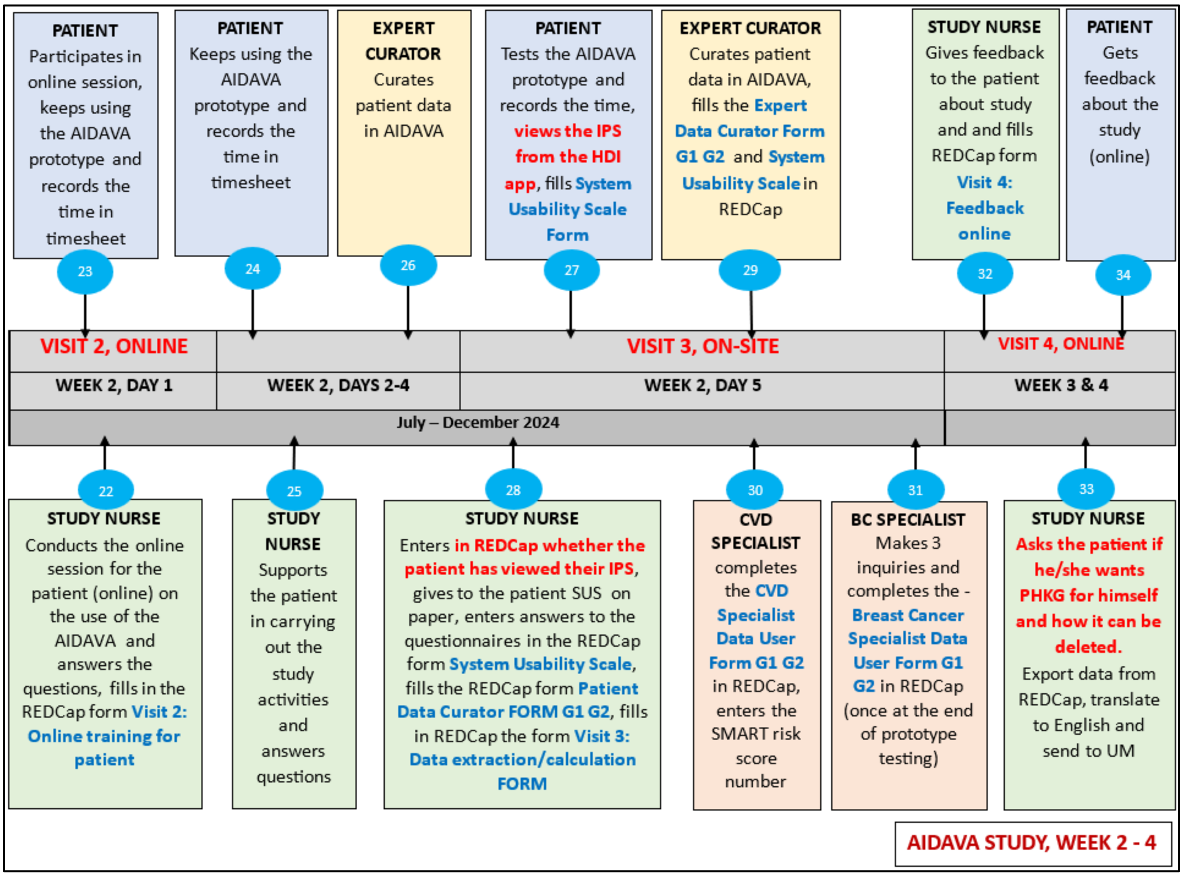

Supplementary Figure 3. *Description of the different steps to test the AIDAVA system with patients (Weeks 2 to 4)*
