# Supplementary material for: An AI-powered data curation and publishing virtual assistant: usability and explainability/causability of, and patient interest in the first-generation prototype
Source: Front Digit Health. 2025 Oct 17;7:1629413. doi: 10.3389/fdgth.2025.1629413 (PMC12576891; doi:10.3389/fdgth.2025.1629413)
Supplement: Supplementary file 2 [file Datasheet2.pdf]

Supplementary Table 1 *List of curation tools within the library of the G1 prototype*

Curation tool	Software used
Ingestion Data Validator	Developed de novo
Mapping Tool Editor	Developed de novo
Extensions of OpenRefine	OpenRefine
Optical Character Recognition	Tesseract
Entity Linking	SNOMEDICO, AI4EU-CODE, Onto Entity Matching
MutateAndTransform	OpenRefine, RML Mapper, YARRRML parser
Averbis Health Discovery	Health Discovery
NLP to KG	Developed de novo
Precision Medicine Toolbox	Open-source Python package

Supplementary Table 2 *Overview of the amount of documents processed, ingested and curated*

**Overview of processed data sources/files (extracts from the system)**				
	**NEMC**	**MUG**	**MUMC**	**Maastro**
**# processed**	763	327[(2)]	546	191[(4)]
**Comparison against total # processed**				
**# successfully ingested**	537[(1)] (70%)	307 (94%)	521 (97%)	97 (51%)
**# failed during ingestion**	226 (30%)	20 (6%)	15 (3%)	94 (49%)
**# did not start curation (no request)**	4 (1%)	62 (19%)	0 (0%)	0 (0%)
**# failed curation**	19	32	464[(3)]	97
**# with open questions**	196	17	30	0
**Comparison against total # successful ingested**				
**# completed curation**	318 (59%)	196 (64%)	27 (5%)[(3)]	0 (0%)
**# automatically curated**	250 (47%)	127 (41%)	19 (4%)[(3)]	0 (0%)
**# which required human intervention**	264 (49%)	86 (28%)	31 (6%)[(3)]	0 (0%)

(1) NEMC has a low % of ingested documents as they were the first site with more errors at the beginning, that have been fixed, but the ingestion has not been restarted after the fix was applied.
(2) The number of documents in MUG is lower than in the other sites because 7 out of the 11 BC patients did not upload hospital files. 4 out of 11 BC patients curated in AIDAVA 60 csv files from the hospital and 57 files from MIDATA;. 7 CVD patients curated in AIDAVA 154 csv files from the hospital and 53 files from MIDATA
(3)The first eight patients could both ingest and curate data, the last six were only able to ingest without curating because of configuration issues that could not be solved by the end of December; this also explain the low percentage in the last 3 row for MUMC
(4) Maastro patients ingested data but curation was not possible because of configuration issues explained before; these issues were solved end December at a time it was difficult to ask patients to re-assess the system.

Supplementary Table 3 *Documents automatically curated and needing HITL*

Metric 2.3 - Number of documents curated automatically (extracts from the system)						
	NEMC		MUG		MUMC	Maastro
	CVD	BC	CVD	BC	CVD	BC
Number patients active	23		18		14	15
# data sources/files successful ingested	537		307		521	97
Average # documents per patient	23,3		17		37,5	0
M2.3. # documents curated automatically	250 (47%)		127 (41%)		19 (4%)	0 (0%)
Documents that require human intervention(1)	265 (49%)		86 (28%)		31 (6%)	0 (0%)
M2.4. questions answered by patient(2)	1036		143		52	0
M2.5. questions sent to the curators	1212		214		13	0
M2.6. questions not answered by curator (3)	257		164		1	0
Average number of questions per document	4,9		2,6		0.2	0

(1) Patients were asked the following questions

- check & correct OCR-extracted text (in most cases it was not feasible to answer this question, as it would have required typing the whole text of the medical document manually...)
- missing datetime (most frequent question, over 90% of questions were of this type)
- missing coding system and version for "known absent - code" or "negative - code" (this question is not understandable and cannot be answered neither by patient nor by curator)

(2) Many of the answers to the questions were nonsensical: since patients were annoyed/frustrated by the type of non-answerable questions of the system (e.g. when the system asked to check an OCR-extracted text which was completely nonsense or which simply wasn't there) and in those cases they often just typed in something and checked "Yes, this is correct"

(3) There was a high number of questions that even the curators could not answer; this depicts the fact that a lot of AIDAVA's questions were not understandable (e.g. when the system asked for "coding system and version of known absent - code") or not feasible to answer (e.g. when the system asks the user to check and correct an OCR-extracted text that is completely nonsense).

(3) This average number is comparatively low, since for all photos of medical documents which patients uploaded to MIDATA, there was (initially) only 1 question, namely to check and correct the OCR-extracted text. Only after patients typed a meaningful text themselves, further questions of the system were raised. However, this was rarely the case for these documents uploaded as a photo, as it is not feasible to type the complete text of a medical report manually - especially on a smartphone

Supplementary Table 4 *Data elements included in the BC registries*

**Data item**	**Data output**
**Patient data**	
Patient ID	
Age at diagnosis	# years
Sex	Male/Female
Prior ipsilateral BC	YES/NO/UNKNOWN
**Baseline tumour factors**	
Lesion ID	
Radiological tumor size	mm
Laterality	LIST[a]
Clinical T-stage	LIST
Clinical N-stage	LIST
Clinical M-stage	LIST
Date of diagnosis	dd-mon-yyyy
**Diagnostic procedure and results**	
Number of nodes resected	NUMBER

Number of involved nodes	NUMBER
Conclusion lymph node mapping	LIST
**Pathology repost (excision specimen)**	
Date of diagnosis invasive / insitu component	dd-mon-yyyy
Invasive beast cancer / invasive component	
Histology invasive breast cancer	LIST
Size invasive component	mm
Topography	LIST
Grade of differentiation invasive cancer	LIST
Minimal resection margins invasive breast cancer	LIST
Progesterone receptor	Positive/Negative/Not tested/ Unknown
Oestrogen receptor	Positive/Negative/Not tested/ Unknown
HER2 receptor	Positive/Negative/Not tested/ Unknown
In situ cancer / in situ component	
Histology in situ component	LIST
Grade of differentiation in situ component	LIST
Size of DCIS	mm
Minimal resection margins DCIS	LIST

Other items	
pT-stage	LIST
ypT-stage	LIST
pN-stage	LIST
ypN-stage	LIST
pM-stage	LIST
**Surgery and pathology**	
Surgery primary tumour	
Date of surgery primary tumour	dd-mon-yyyy
Type of surgery	LIST
Re-resection	YES/NO
If yes, date of re-resection	dd-mon-yyyy
Breast reconstruction	LIST
Axillary lymph node dissection	
Axillary lymph node dissection performed	YES/NO
If yes, Date of axillary lymph node dissection	dd-mon-yyyy
**Chemotherapy and other systemic treatment**	
Chemotherapy	

Neoadjuvant chemotherapy	YES/NO/UNKNOWN
Date start neoadjuvant chemotherapy	dd-mon-yyyy
Adjuvant chemotherapy	YES/NO/UNKNOWN
Date start adjuvant chemotherapy	dd-mon-yyyy
Hormonal therapy	
Neoadjuvant endocrine therapy	YES/NO/UNKNOWN
Date start neoadjuvant hormonal therapy	dd-mon-yyyy
Adjuvant endocrine therapy	YES/NO/UNKNOWN
Date start adjuvant endocrine therapy	dd-mon-yyyy
Targeted / immunotherapy	
Neoadjuvant anti HER2 therapy	YES/NO/UNKNOWN
Date start neoadjuvant anti HER2 therapy	dd-mon-yyyy
Adjuvant anti HER2 therapy	YES/NO/UNKNOWN
Date start adjuvant anti HER2 therapy	dd-mon-yyyy
**Radiotherapy related factors**	
Mamma specific radiotherapy related factors	
Neoadjuvant radiotherapy	YES/NO/UNKNOWN
Start neoadjuvant radiotherapy	dd-mon-yyyy

Adjuvant radiotherapy	YES/NO/UNKNOWN
Start adjuvant radiotherapy	dd-mon-yyyy
Sequence of radiotherapy	LIST
Target volume: Breast	YES/NO
Target volume: Partial breast	YES/NO
Target volume: Chest wall	YES/NO
Target volume: Level 1&2	YES/NO
Target volume: Level 3&4	YES/NO
Target volume: Interpectoral nodes	YES/NO
Target volume: Internal mammary nodes	YES/NO
Total dose prescribed	Gy
Number of fractions to elective regions	number
If boost, Fraction dose boost	Gy
If boost, Number of fractions to boost regions	number
After treatment delivery	
Date of start RT	dd-mon-yyyy
Date of last RT	dd-mon-yyyy

Radiation-induced side effects

Dermatitis	YES/NO/UNKNOWN
Lymphedema	YES/NO/UNKNOWN
Rib fracture	YES/NO/UNKNOWN
Heart toxicity	YES/NO/UNKNOWN
Radiation Pneumonitis	YES/NO/UNKNOWN
Comorbidity items	
Hypertension	YES/NO/UNKNOWN
Smoking	YES/NO/UNKNOWN
Diabetes	YES/NO/UNKNOWN
BMI	number
**Tumour follow up**	
Date of last follow up	dd-mon-yyyy
Status at last follow up	LIST
In case of status 1 - 6	
Local recurrence	YES/NO
If YES, date of local recurrence	dd-mon-yyyy
Regional recurrence	YES/NO
If YES, date of regional recurrence	dd-mon-yyyy

Distant metastases	YES/NO
If YES, date of distant recurrence	dd-mon-yyyy
**Tumour screening**	
Postoperative multi-disciplinary team meeting	dd-mon-yyyy
Preoperative multi-disciplinary team meeting	dd-mon-yyyy
Was cancer found in screening?	YES/NO
Is patient receiving palliative treatment?	YES/NO
Is patient enrolled in registred trial?	YES/NO

[a] LIST refers to a subset of data elements, which vary according to the data item

Supplementary Table 5 *Variables used in calculating the SMART risk score*

**Data description**	**Data Type**	**Values**
Age	REAL/INTEGER	in years
Sex	BOOLEAN (Y or N)	1 = Male, 0 = Female
Smoking status	BOOLEAN (Y or N)	1 = True , 0 = False
Systolic blood pressure	INTEGER (3 digit)	in mmHg (if needed transform)
Diabetes diagnosis	BOOLEAN (Y or N)	1 = True , 0 = False
Coronary artery disease	BOOLEAN (Y or N)	1 = True , 0 = False
Cerebrovascular disease	BOOLEAN (Y or N)	1 = True , 0 = False
Aortic aneurysm	BOOLEAN (Y or N)	1 = True , 0 = False
Peripheral artery disease	BOOLEAN (Y or N)	1 = True , 0 = False
yearsSinceFirstDiagnosis	Integer	1 = True , 0 = False
High-Density Lipoprotein (HDL) cholesterol	Number (value)<br>STRING (unit)	in mmol/l
Total cholesterol	Number (value)<br>STRING (unit)	in mmol/l
Estimated glomerular filtration rate	Number (value)<br>STRING (unit)	in mL/min/1.73m²
Creatinine	Number (value)	in mmol/l

	STRING (unit)	
C-reactive protein	Number (value)<br>STRING (unit)	in mg/L
Anticoagulation use	BOOLEAN (Y or N)	1 = True , 0 = False

Supplementary Tab 6 *Health and Digital literacy questionnaire (self-drafted)*

**Health literacy**	
Did you receive any medical training or education?	No education (0) --- Expert education (5)
I often need someone to help me (or need to do some internet research) with reading hospital materials	Always (0) --- Never (5)
I often face difficulties learning about my / a patient's medical condition	Always (0) --- Never (5)
I know how to find reliable information about medical terms on the internet	Strongly disagree (0) --- Strongly agree (5)
I am familiar with different units of measurement in the medical context and know their differences (e.g. mHg and cmHg)	Strongly disagree (0) --- Strongly agree (5)
I am familiar with medical standards and terminologies, such as ICD and SNOMET CT	Strongly disagree (0) --- Strongly agree (5)
**Digital literacy**	
Did you receive any computer science or data science training or education?	No education (0) --- Expert education (5)
I know how to access the metadata of an electronic file	Strongly disagree (0) --- Strongly agree (5)
I am always keen on understanding the technical details of innovative solutions	Strongly disagree (0) --- Strongly agree (5)
When I use a search engine, I can take advantage of its advanced features	Strongly disagree (0) --- Strongly agree (5)
I know how to protect myself from unwanted and malicious online encounters and materials	Strongly disagree (0) --- Strongly agree (5)

When I face a technical problem, I am able to find solutions on the internet	Strongly disagree (0) --- Strongly agree (5)
